# Determinants of acute and subacute case-fatality in elderly patients with hypertensive intracerebral hemorrhage

**DOI:** 10.1016/j.heliyon.2023.e20781

**Published:** 2023-10-06

**Authors:** Zhao-Ying Zhu, Li-Fang Hao, Li-Chuan Gao, Xiao-Long Li, Jie-Yi Zhao, Tao Zhang, Gui-Jun Zhang, Chao You, Xiao-Yu Wang

**Affiliations:** a Department of Neurosurgery, West China Hospital, Sichuan University, No. 37 Guo Xue Xiang, Chengdu, 610041, Sichuan, China; b Department of Radiology, Liaocheng The Third People's Hospital, Liaocheng, China; c Operating Room, West China Hospital, Sichuan University/West China School of Nursing, No. 37 Guo Xue Xiang, Chengdu, 610041, Sichuan, China

**Keywords:** Elderly, Hypertensive intracerebral hemorrhage, Mortality, Morbidity, Nomogram

## Abstract

**Background:**

Given that limited reports have described the survival and risk factors for elderly patients with hypertensive intracerebral hemorrhage (HICH), we aimed to develop a valid but simple prediction nomogram for the survival of HICH patients.

**Methods:**

All elderly patients ≥65 years old who were diagnosed with HICH between January 2011 and December 2019 were identified. We performed the least absolute shrinkage and selection operator (Lasso) on the Cox regression model with the R package glmnet. A concordance index was performed to calculate the nomogram discrimination; and calibration curves and decision curves were graphically evaluated by depicting the observed rates against the probabilities predicted by the nomogram.

**Results:**

A total of 204 eligible patients were analyzed, and over 20 % of the population was above the age of 80 (65–79 years old, n = 161; 80+ years old, n = 43). A hematoma volume ≥13.64 cm3 was associated with higher 7-day mortality (OR = 6.773, 95 % CI = 2.622–19.481; p < 0.001) and higher 90-day mortality (OR = 3.955, 95 % CI = 1.611–10.090, p = 0.003). A GCS score between 13 and 15 at admission was associated with a 7-day favorable outcome (OR = 0.025, 95 % CI = 0.005–0.086; p < 0.001) and a 90-day favorable outcome (OR = 0.033, 95 % CI = 0.010–0.099; p < 0.001).

**Conclusions:**

Our nomogram models were visualized and accurate. Neurosurgeons could use them to assess the prognostic factors and provide advice to patients and their relatives.

## 1 Introduction

Stroke is a major cause of mortality and disability worldwide [1]. Hypertensive intracerebral hemorrhage (HICH) is a type of stroke of the central nervous system, accounting for approximately 30%–60 % of ICH [2], with a high mortality in 36%–43 % of patients and a high morbidity in more than 90 % of patients [3]. In recent years, many studies have reported variable frequencies regarding hypertension among all-cause ICH in elderly individuals, ranging from 51.8% to 86.6 % [[4], [5], [6], [7], [8]]. Previous studies demonstrated that stroke mortality and morbidity increased with age, and an increasing number of patients suffer from a steadily high rate of mortality due to population aging [9]. In addition, elderly patients have a 10-fold increase in ICH risk compared with younger adults [10].

Some studies have evaluated risk factors and survival in HICH patients; however, limited literature in which stratification of HICH patients by age was performed. Additionally, these predictions of HICH elderly patients have not been identified. Hence, an individual prediction model is imperative to evaluate the prognosis of elderly patients. Nomograms have been widely used as predictive models that calculate different variables and quantify clinical event risks [11,12]. Moreover, evaluating ICH outcome on day 7 contributed to understand the mechanism underlying neurological function changes [[13], [14], [15]]. Therefore, in this study, we aimed to establish a valid but simple tool for the probability of morbidity and mortality at 7 days and 90 days in HICH elderly patients aged ≥65 years within 6 h from onset.

## 2 Materials and methods

Elderly patients were defined as those aged ≥65 years per the definition established by the World Health Organization [16]. All elderly patients diagnosed with HICH between January 2011 and December 2019 were identified. The Institutional Review Board of West China Hospital approved this study. ICH was diagnosed by computer technology (CT) and other clinical symptoms: headache, vomiting, limb weakness, and unconsciousness. The diagnostic criteria of HICH included both of the following conditions: 1) a history of hypertension and 2) the typical hemorrhage locations: ventricles, basal ganglia, thalamus, brain stem and cerebellar hemisphere. The patient's data were recorded in our electronic medical system. In this research, elderly patients who were diagnosed with HICH had an identified hypertension history were included in this cohort. The exclusion criteria included one of the following: 1) more than 6 h after ictus, 2) hematoma located in the lobe(s), or 3) hematoma due to causes other than hypertension.

Outcomes were dichotomized to short-term 7-day mortality and long-term 90-day mortality and morbidity. Stroke severity was assessed using the Glasgow Coma Scale (GCS) score and modified Rankin Scale (mRS) score. Neurological examination was routinely performed on a daily basis after admission. The GCS score was divided into four parts: 3–5, 6–8, 9–12, and 13–15. The study's endpoint was the neurological outcome at 7 days and 90 days (mRS score≤2 as a good outcome and mRS score>2 as a poor outcome).

An axillary temperature ≥37.5 °C was defined as hyperthermia. ICH hematoma volume was calculated by using the ABC/2 method. Hematoma location was defined as follows: 1) lobe, dominantly invasive of the cortical/subcortical white matter of the cerebral lobes hematoma, and 2) deep location, dominantly invasive of the basal ganglia and/or thalamus hematoma. Then, locations in this study were divided into 4 parts: ventricles, cerebellum, deep location, and brain stem. Surgical history was defined as non–ICH–related surgical experiences. Cerebral infarction, cerebral atrophy, and demyelination in white matter were estimated by CT at admission. Surgery strategies for ICH mainly included open craniotomy and minimally invasive catheter evacuation. Cutoff points for laboratory factors were mainly based on our laboratory test criteria.

### 2.1 Statistical analysis

We performed the least absolute shrinkage and selection operator (Lasso) on the Cox regression model with the R package glmnet, and the tenfold cross-validation approach was used to identify the optimal parameter λ. All statistical tests were performed using R (version 3.6.0, https://www.r-project.org) and SPSS software (version 25.0; IBM Corp., Armonk, New York, USA). The features were considered odds ratios (ORs) with 95 % confidence intervals (CIs), and P values < 0.05 with statistical significance were used to construct the nomogram using the R rms package. The Lasso model for 7-day and 90-day mortality included 28 features: gender, age, season, surgical history, cerebral infarction, diabetes, alcohol habit, smoking habit, marital status, temperature, GCS score at admission, mRS score at admission, location, ventricles involved, hematoma volume, plate, white blood cell (WBC), neutrophilic granulocyte lymphocyte ratio (NLR), international normalized ratio, glucose, uric acid, triglyceride, cholesterol, high-density lipoprotein, low-density lipoprotein, systolic blood pressure (SBP), diastolic blood pressure (DBP), and L-lactic dehydrogenase. A concordance index (C-index) was performed to calculate the nomogram discrimination by a bootstrap method with 1000 resamples, and calibration curves were graphically evaluated by depicting the observed rates against the probabilities predicted by the nomogram. In addition, the discriminatory capabilities of the nomogram were assessed with the area under the receiver operating characteristic curve (AUC) and decision curve analysis (DCA).

## 3 Results

From a single institute, as an estimated 581 patients were diagnosed with HICH, a total of 204 eligible patients were analyzed, and over 20 % of the population was above the age of 80 (65–79 years old, n = 161; 80+ years old, n = 43). The demographic, radiologic, and laboratory characteristics of the study population are summarized in Table 1. The mean time±standard deviation (SD) from onset of this population was 4.3 ± 1.2 h, and the median time of hospital stay was 5 days. Male patients comprised a larger proportion of the cohort than female patients, with a male/female ratio of 1.76:1. The most common clinical symptoms were limb weakness (n = 105, 51.5 %), consciousness disorder (n = 91, 44.6 %), headache (n = 65, 31.9 %), dizziness (n = 51, 25.0 %), and speech disorder (n = 46, 22.5 %). In terms of location, 39.7 % (n = 81) of cases of HICH were in the basal ganglia, 24.5 % of cases (n = 50) were in the thalamus, 13.7 % of cases (n = 28) were in the brain stem, and 11.3 % of cases (n = 23) were in the cerebellum.

**Table 1:** Demographic characteristics of 204 elderly patients with hypertensive intracranial hemorrhage.

Variable		Total, n (%)	
		204	
Sex			
Male		130 (63.7)	
Female		74 (36.3)	
Time from onset, hours			
Mean ± SD		4.3 ± 1.2	
Season			
Spring		47 (23.0)	
Summer		47 (23.0)	
Autumn		53 (26.0)	
Winter		57 (27.9)	
Symptoms			
Headache		65 (31.9)	
Dizzy		51 (25.0)	
Limbs weakness		105 (51.5)	
Sensory disturbance		15 (7.4)	
Speech disorder		46 (22.5)	
Feces and urine incontinence		35 (17.2)	
Consciousness disorder		91 (44.6)	
Epilepsy		3 (1.5)	
Surgical history		59 (28.9)	
Hospital stay, days			
Median		5	
Marital status			
Unmarried		44 (21.6)	
Smoking habit		67 (32.8)	
Alcohol habit		48 (23.5)	
History of diabetes		33 (16.2)	
History of heart disease		22 (10.8)	
Cerebral infarction		35 (17.2)	
Cerebral atrophy		18 (8.8)	
Demyelination in white matter		18 (8.8)	
Lung infection			
Yes		18 (8.8)	
Unknown/no		186 (91.2)	
Location			
Basal ganglia		81 (39.7)	
Thalamus		50 (24.5)	
Basal ganglia + thalamus		12 (5.9)	
Brain stem		28 (13.7)	
Primary ventricle		10 (4.9)	
Cerebellum		23 (11.3)	
Ventricle involved			
No		67 (32.8)	
Yes		137 (67.2)	
Volume, cm^3^			
<12.5		102 (50)	
≥12.5		102 (50)	
Temperature at admission, °C			
<37.5		194 (95.1)	
≥37.5		10 (4.9)	
GCS admission			
3-5		45 (22.1)	
6-8		33 (16.2)	
9-12		33 (16.2)	
13-15		93 (45.6)	
Therapy			
No		202 (99.0)	
Surgery		2 (1.0)	
SBP, mm/Hg			
<140		19 (9.3)	
140-159		35 (17.2)	
160-179		60 (29.4)	
180-199		43 (21.1)	
≥200		47 (23.0)	
DBP, mm/Hg			
<90		72 (35.3)	
90-99		57 (27.9)	
100-109		34 (16.7)	
110-119		21 (10.3)	
≥120		20 (9.8)	
White blood cell, 10^9/L			
≤10		85 (41.7)	
>10		119 (58.3)	
Glucose, mmol/L			
<5.9		35 (17.2)	
≥5.9		169 (82.8)	
Uric acid, umol/L			
≤380		155 (76.0)	
>380		49 (24.0)	
LDH, IU/L			
≤250		158 (77.5)	
>250		46 (22.5)	
Platelets, 10^9/L			
<100		44 (21.6)	
≥100		160 (78.4)	
INR			
<1.16		171 (83.8)	
≥1.16		33 (16.2)	
Triglyceride, mmol/L			
<1.84		162 (79.4)	
≥1.84		42 (20.6)	
Cholesterol, mmol/L			
≤5.7		178 (87.3)	
>5.7		26 (12.7)	
HDL, mmol/L			
≤0.9		20 (9.8)	
>0.9		184 (90.2)	
LDL, mmol/L			
≤4		197 (96.6)	
>4		7 (3.4)	
NLR			
Mean ± SD		11.1 ± 9.0	
7-day mortality		55 (27.0)	
90-day mortality		73 (35.8)	

DBP Diastolic blood pressure, GCS Glasgow Coma Scale, HDL High-density lipoprotein, INR International normalized ratio, LDH Lactic dehydrogenase, LDL Low-density lipoprotein, NLR neutrophilic granulocyte/lymphocyte ratio, SBP Systolic blood pressure.

The common clinical presentations were smoking habit (n = 67, 32.8 %), alcohol habit (n = 48, 23.5 %), diabetes history (n = 33, 16.2 %), heart disease history (n = 22, 10.8 %), cerebral infarction (n = 35, 17.2 %), cerebral atrophy (n = 18, 8.8 %), and demyelination in white matter (n = 18, 8.8 %). Surgical removal of hematoma was undertaken in only 2 (1.0 %) cases. The frequencies of high hypertension (SBP ≥200 mm/Hg) and poor admission status (GCS score 3–5) were relatively high (n = 47, 23.0 %; n = 45, 22.1 %). Optimal laboratory feature thresholds were mainly based on our institute criteria.

**Fig. 1:**
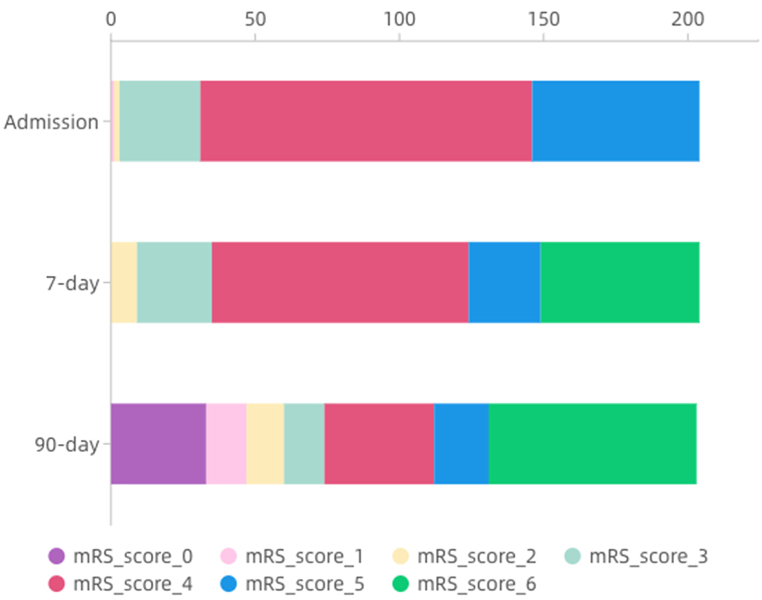
Distribution and changes in mRS scores at different times.

The mean and median mRS scores at the time of admission were 4.1 and 4 (range 1–5; score 1, n = 1 (0.5 %); score 2, n = 2 (1.0 %); score 3, n = 28 (13.7 %); score 4, n = 115 (56.4 %); score 5, n = 58 (28.4 %)), respectively. Compared to the mRS scores at admission, the scores of 25 patients improved, 104 patients worsened, 20 patients remained stable, and 55 patients died at 7 days. With respect to morbidity and mortality at 90 days, 33 patients had mRS scores of 0, 27 had scores of 1–2, 71 had scores of 3–5, and 72 died (Fig. 1).

**Fig. 2:**
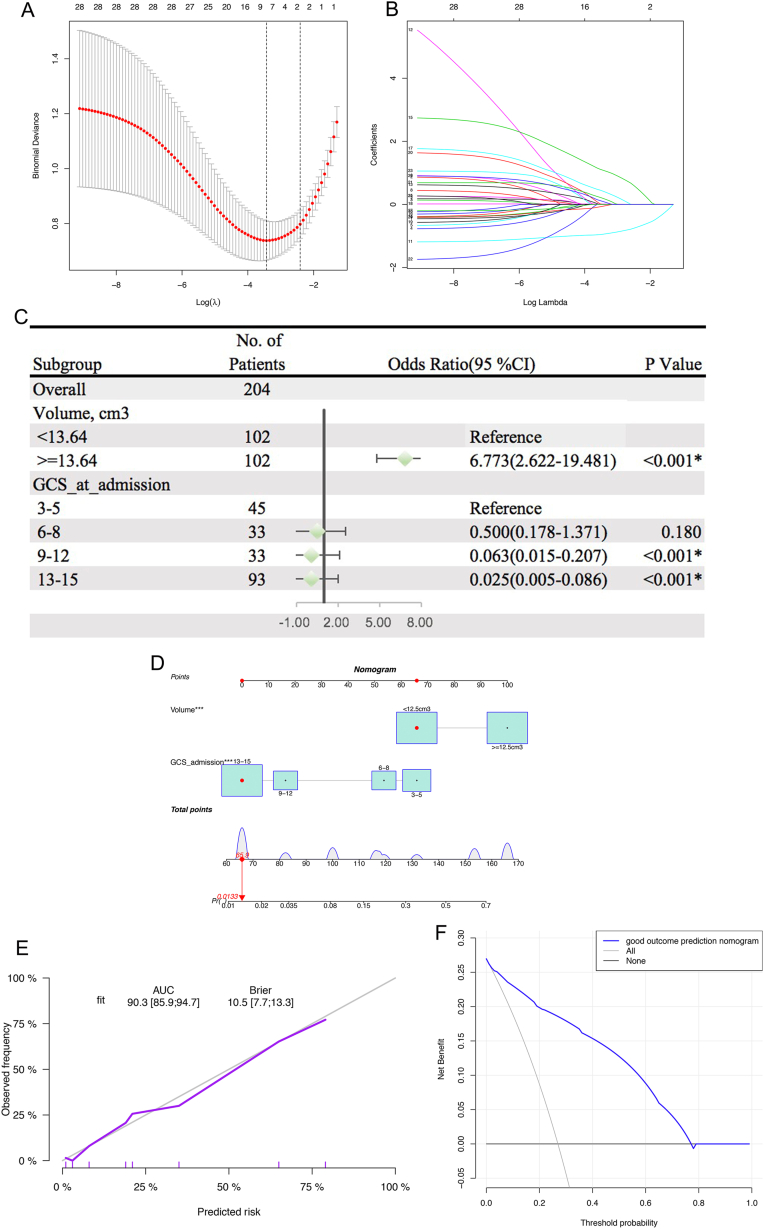
Lasso model. A.B Tenfold cross-validation for tuning parameter selection in the lasso model C Forest plot showing ORs for 7-day mortality in elderly patients with hypertensive intracerebral hemorrhage D Nomogram predicting 7-day mortality probability E.F Calibration curve and decision curve for the nomogram.

**Fig. 3:**
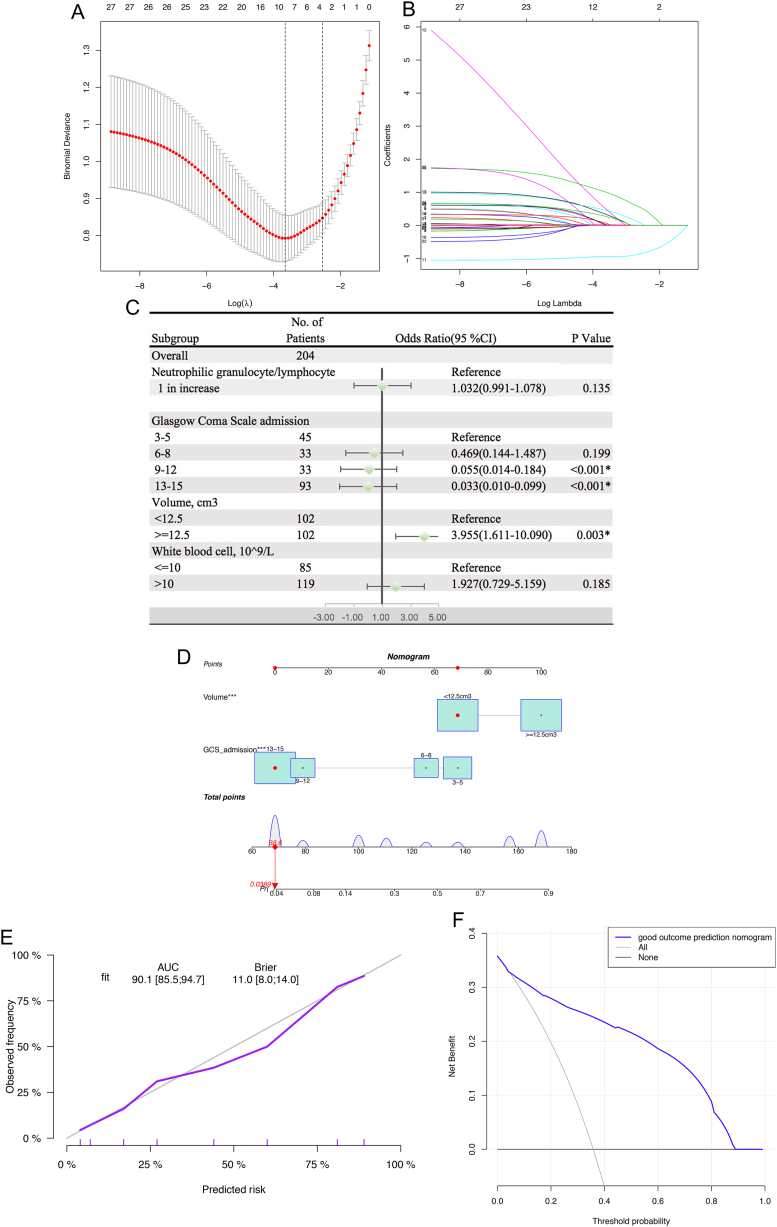
Lasso model. A.B Tenfold cross-validation for tuning parameter selection in the lasso model C Forest plot showing ORs for 90-day mortality in elderly patients with hypertensive intracerebral hemorrhage D Nomogram predicting 90-day mortality probability E.F Calibration curve and decision curve for the nomogram.

GCS score at admission and hematoma volume were selected for 7-day mortality (Fig. 2A and B); GCS score at admission, hematoma volume, NLR, and WBC count were selected for 90-day mortality (Fig. 3A–B); and only GCS score at admission was selected for 90-day neurological outcome. After adjusting for the confounding effects of variables, our multivariate analysis revealed that a hematoma volume ≥13.64 cm3 was associated with higher 7-day mortality (OR = 6.773, 95 % CI = 2.622–19.481; p < 0.001) and higher 90-day mortality (OR = 3.955, 95 % CI = 1.611–10.090, p = 0.003). Likewise, a GCS score between 13 and 15 at admission was associated with a 7-day favorable outcome (OR = 0.025, 95 % CI = 0.005–0.086; p < 0.001) and a 90-day favorable outcome (OR = 0.033, 95 % CI = 0.010–0.099; p < 0.001) (Fig. 2, Fig. 3C). Higher NLR (1-fold increase) and WBC (10*10^9^/L) count remained significant in the Lasso model, but this effect failed in multivariate logistic regression analysis. The nomogram model that incorporated the above independent factors was developed and presented (Fig. 2, Fig. 3D). The C-index and AUC for the 7-day prediction nomograms were 0.903 (95 % CI = 0.859–0.947) and 90.3 (95 % CI = 85.9%–94.7 %), respectively (Fig. 2E), and the C-index and AUC for the 90-day prediction nomograms were 0.901 (95 % CI = 0.855–0.947) and 90.1 (95 % CI = 85.5%–94.7 %), respectively (Fig. 3E). The calibration curve (Fig. 2, Fig. 3E) and DCA (Fig. 2, Fig. 3F) indicated the good ability of the nomogram.

## 4 Discussion

We identified 204 elderly cases of HICH between 2011 and 2019, assuming that 35.1 % (204/581) of HICH patients were elders based on our institutional experience, which was lower than that in previous studies; in addition, more than 20 % of cases were very elderly patients ≥80 years old. Only a few retrospective studies on HICH patients aged ≥65 years have been reported to date. Given the unclearly limited reports about these HICH elderly patients, we performed a population-based analysis to identify that hematoma volume and GCS score at admission were associated with outcome, and then a nomogram with a good prediction was developed for elderly patients with HICH.

The investigators found that very elderly patients with ICH were more commonly female than male [5,7,17]. In contrast, male patients were more subject to HICH events than female patients in our study. This difference in sex, indeed, may have reflected true biological features, as varied causes led to ictus. Previous studies reported that ICH in winter had a peak incidence [18,19]. This finding was also observed in our study. Basal ganglia-thalamus hemorrhage in patients aged 60–70 years is regarded as a fatal end stage of hypertension [20]. However, we did not find a significant relationship between location and survival. Based on this Chinese elderly cohort with HICH, we found that there was no significant interaction between SBP or DBP and outcome. There were also some studies showing that elevated blood pressure did not increase the risk for hemorrhagic stroke [[20], [21], [22]].

More importantly, in HICH patients, poor functional outcome was independently correlated with larger hematoma volume and lower GCS score. Additionally, in multiple studies, a larger hematoma volume and lower GCS score were associated with increased morbidity and mortality [20,[23], [24], [25]].

Hypertension is considered a chronic inflammatory disease, and many studies have tried to find an association between hypothermia and inflammation [[26], [27], [28]]. Fever is a common complication that occurs in one-third of patients with ICH and is associated with poor outcome [29]. A large population-based cohort showed that 148 of 795 patients developed a temperature greater than 37.5 °C within the first 24 h of hospital admission and it indicated poor outcome in those with fever [30]. However, we failed to obtain duplicated results. Limited cases in our studies showed a high temperature ≥37.5 °C. In addition, in the Lasso model, the higher NLR and WBC count were associated with high 90-day mortality, but the multivariate analysis for these two factors did not reveal significance, while prior literature advocated for the association between NLR [[31], [32], [33]] or WBC [34,35] and mortality. However, to the best of our knowledge, the relations between these two factors and prognosis in elderly patients with ICH have not been described [4,5,7,17]. A prospectively large study would provide more robust evidence.

Although recent years have observed advances in neuro-diagnostic and treatment strategies, treatment planes using or not using surgery have been debated due to surgical complications or mortality in elderly patients. There have been a few studies attempting to verify the safety and efficacy of surgery in ICH [2,36,37]. Minimally invasive surgeries have attracted the attention of many scholars. In a retrospective cohort of 51 patients, Zhang et al. described that 41.2 % (n = 21) of patients received neuroendoscopic surgery as a minimally invasive treatment and 58.8 % of patients (n = 30) received craniotomy. The authors found that minimally invasive surgery was capable of efficacy and safety and provided good functional recovery [38]. A striking finding in our study was the limited use of surgery in elderly patients over a decade. In addition, hemorrhage in lobe location may have a major response for rare cases with surgical removal. Our findings confirmed a previous report and suggested a lower incidence rate of surgery in elderly patients [17]. Elderly patients are more vulnerable to rebleeding and pulmonary infection, which are dominantly fatal complications. Further prospective analysis is imperative to propose optimal treatment paradigms for elderly patients with HICH.

## 5 Limitations

While we tried our best to select many clinical factors affecting the morbidity and mortality of elderly patients with HICH in this retrospective study, it was possible that other confounding variables were not incorporated into the Lasso analysis. Unfortunately, we did not solve the problem of finding optimal treatment strategies in HICH elderly patients. Additionally, large population-based studies regarding age are needed to estimate the epidemiology, treatment strategies and survival in the hope of better understanding and improving the survival of elderly patients with HICH.

## 6 Conclusions

Based on our analysis, a high GCS score at admission and a small hematoma volume were significantly favorable factors related to both short- and long-term outcomes. Our nomogram models were readily visualized and accurate, and neurosurgeons could use them to assess the prognostic factors and provide advice to patients and their relatives. Prospective studies may be performed to propose useful treatment strategies.

## Ethics approval

This study was approved by the Biomedical Ethics Committee of 10.13039/501100013365West China Hospital of Sichuan University (NO.2020–208).

## Data availability statement

The data supporting the findings of this study are available from the corresponding authors upon reasonable request.

## Informed consent

Informed consent was obtained from all patients in our institute.

## Funding

Supported by 10.13039/501100010822Chengdu Science and Technology Bureau project (NO. 2019-YF05-00333-SN), 1.3.5 project for disciplines of excellence, 10.13039/501100013365West China Hospital, Sichuan University (NO. 2018HXFH010), and Sichuan Science and Technology Program (NO•2020YFS0091).

## CRediT authorship contribution statement

**Zhao-Ying Zhu:** Conceptualization, Data curation, Formal analysis, Methodology, Project administration, Writing – original draft, Writing – review & editing. **Li-Fang Hao:** Conceptualization, Data curation, Formal analysis, Methodology, Supervision, Validation, Writing – original draft, Writing – review & editing. **Li-Chuan Gao:** Conceptualization, Data curation, Formal analysis, Methodology, Writing – original draft, Writing – review & editing. **Xiao-Long Li:** Conceptualization, Data curation, Formal analysis, Investigation, Methodology, Project administration, Resources, Software, Writing – original draft. **Jie-Yi Zhao:** Conceptualization, Formal analysis, Investigation, Methodology, Project administration, Resources, Software, Supervision, Validation, Visualization. **Tao Zhang:** Formal analysis, Funding acquisition, Investigation, Methodology, Project administration, Resources, Software. **Gui-Jun Zhang:** Conceptualization, Data curation, Investigation, Methodology, Project administration, Software, Validation, Writing – original draft. **Chao You:** Funding acquisition, Investigation, Resources, Software, Supervision, Validation, Writing – original draft. **Xiao-Yu Wang:** Conceptualization, Funding acquisition, Investigation, Methodology, Resources, Supervision, Writing – original draft, Writing – review & editing.

## Declaration of competing interest

The authors declare that they have no known competing financial interests or personal relationships that could have appeared to influence the work reported in this paper.
